# Randomized study of evolocumab in patients with type 2 diabetes and dyslipidaemia on background statin: Pre‐specified analysis of the Chinese population from the BERSON clinical trial

**DOI:** 10.1111/dom.13700

**Published:** 2019-04-14

**Authors:** Yundai Chen, Zuyi Yuan, Juming Lu, Freddy G. Eliaschewitz, Alberto J. Lorenzatti, Maria Laura Monsalvo, Nan Wang, Andrew W. Hamer, Junbo Ge

**Affiliations:** ^1^ Department of Cardiology Chinese People's Liberation Army General Hospital Beijing China; ^2^ First Affiliated Hospital of Xi'an Jiaotong University Shaanxi China; ^3^ Department of Endocrinology Chinese People's Liberation Army General Hospital Beijing China; ^4^ CPCLIN/DASA ‐ Centro de Pesquisas Clínicas São Paulo Brazil; ^5^ Clinical Research and Cardiology, Instituto Medico DAMIC / Fundación Rusculleda Córdoba Argentina; ^6^ Clinical Development, Amgen Inc. Thousand Oaks California; ^7^ Department of Cardiology, Shanghai Institute of Cardiovascular Diseases Zhongshan Hospital, Fudan University Shanghai China

**Keywords:** dyslipidaemia, evolocumab, hyperlipidaemia, phase 3, type 2 diabetes

## Abstract

**Aim:**

The aim of this study was to evaluate the efficacy and safety of evolocumab with background atorvastatin in Chinese patients with type 2 diabetes mellitus (T2DM) and hyperlipidaemia or mixed dyslipidaemia.

**Materials and methods:**

This is a pre‐specified analysis of patients in the BERSON study (ClinicalTrials.gov, NCT02662569) in China. Patients initiated background atorvastatin 20 mg/d, after which they were randomized 2:2:1:1 to evolocumab 140 mg every 2 weeks (Q2W) or 420 mg monthly (QM) or to placebo Q2W or QM. Co‐primary endpoints were percentage change in LDL cholesterol (LDL‐C) from baseline to week 12 and from baseline to the mean of weeks 10 and 12. Additional endpoints included atherogenic lipids, glycaemic measures and adverse events (AEs).

**Results:**

Among 453 patients randomized in China, 451 received at least one dose of study drug (evolocumab or placebo). Evolocumab significantly reduced LDL‐C compared with placebo at week 12 (Q2W, −85.0%; QM, −74.8%) and at the mean of weeks 10 and 12 (Q2W, −80.4%; QM, −81.0%) (adjusted *P* < 0.0001 for all) when administered with background atorvastatin. Non‐HDL‐C, ApoB100, total cholesterol, Lp(a), triglycerides, HDL‐C and VLDL‐C significantly improved with evolocumab vs placebo. No new safety findings were observed with evolocumab. The incidence of diabetes AEs was higher with evolocumab compared with placebo. There were no differences over time between evolocumab and placebo in measures of glycaemic control.

**Conclusions:**

In patients in China with T2DM and hyperlipidaemia or mixed dyslipidaemia receiving background atorvastatin, evolocumab significantly reduced LDL‐C and other atherogenic lipids, was well tolerated, and had no notable impact on glycaemic measures.

## 1 INTRODUCTION

China carries the highest diabetes burden of any country worldwide,^1^ with an estimated prevalence of 10.9% in 2013.^2^ Consistent with observations in Western populations, diabetes is a major risk factor for developing cardiovascular disease (CVD) in Chinese adults.^3^ Furthermore, as is the case globally, Chinese patients with type 2 diabetes mellitus (T2DM) have an increased risk of cardiovascular events, including cardiovascular death, myocardial infarction, hypertension and stroke.^4^, ^5^, ^6^, ^7^

Low‐density lipoprotein cholesterol (LDL‐C) is the primary target in the treatment of dyslipidaemia in patients with T2DM,^8^, ^9^, ^10^, ^11^ as elevated LDL‐C has been associated with increased risk of CVD and cardiovascular events.^12^, ^13^, ^14^ Chinese guidelines for the treatment of dyslipidaemia in high‐risk patients with T2DM recommend statin‐based therapy for maintaining LDL‐C below 1.8 mmol/L.^11^ Despite the availability of statin therapy in China and Southeast Asia, attainment of recommended LDL‐C levels has been suboptimal, with 60% to 72% of patients with T2DM in community clinics or hospitals failing to achieve recommended LDL‐C levels.^15^, ^16^, ^17^, ^18^ Observational, pharmacological and pharmacogenetic studies suggest that statin‐related adverse reactions occur at higher rates among Chinese patients than would be observed in a global population.^19^ Suboptimal reduction in LDL‐C among Chinese patients may, therefore, be driven by intolerance to high‐potency high‐dose statin treatment, and under‐utilization may be driven by statin‐related adverse reactions.^19^, ^20^ Consequently, there is an urgent unmet need for novel lipid‐lowering therapies for addition to the maximally‐tolerated statin dose in China.

Analyses from phase 2 and 3 clinical trials have consistently shown that evolocumab, a human monoclonal antibody to proprotein convertase subtilisin/kexin type 9 (PCSK9), significantly reduces LDL‐C without adversely affecting objective measures of glycaemic control in patients with or without diabetes.^21^, ^22^, ^23^, ^24^, ^25^ In the global, randomized, double‐blind, 12‐week phase 3 BERSON study (N = 986), in which approximately half of the patients with T2DM and hyperlipidaemia or mixed dyslipidaemia were enrolled from Chinese centres, efficacy results from the global population showed that evolocumab significantly reduced LDL‐C and improved other lipid levels compared with placebo when administered in combination with background atorvastatin.^26^, ^27^ In the global population, evolocumab was safe and well tolerated, with no notable changes in glycaemic measures throughout the study. This pre‐specified analysis of the BERSON study was designed to investigate the efficacy and safety of evolocumab combined with background atorvastatin in patients at Chinese centres.

## 2 METHODS

### 2.1 Study design, participants, and procedures

BERSON (ClinicalTrials.gov, NCT02662569) was a 12‐week, randomized, double‐blind, placebo‐controlled phase 3 study of subcutaneous evolocumab 140 mg Q2W, evolocumab 420 mg QM, placebo Q2W or placebo QM (2:2:1:1) combined with oral atorvastatin 20 mg/d in patients with T2DM and hyperlipidaemia or mixed dyslipidaemia. Briefly, patients were between 18 and 80 years of age, had been diagnosed with T2DM, were receiving stable pharmacological therapy for diabetes for at least 6 months, had HbAIc of 10% or less and fasting triglyceride levels of 4.5 mmol/L or less (≤400 mg/dL). Patients undergoing statin therapy at screening were obliged to have LDL‐C of at least 2.6 mmol/L (≥100 mg/dL); those not undergoing statin therapy at screening were obliged to have LDL‐C of at least 3.4 mmol/L (≥130 mg/dL). It was required that the lipid‐lowering therapy status of patients remained unchanged for at least four weeks before LDL‐C screening. The study design and rationale, as well as full inclusion and exclusion criteria, were described previously.^26^, ^27^

This prespecified analysis includes only patients who were randomized at centres in China. The study was conducted in accordance with the Guidelines on Good Clinical Practice and with the Ethical Standards for Human Experimentation established by the Declaration of Helsinki. An independent review board or independent ethics committee at each study site reviewed the study and approved the protocol and subsequent amendments to the study protocol. An external, independent data monitoring committee (DMC) periodically reviewed study data, and analyses for the DMC were provided by an independent biostatistical group. All patients provided informed consent before participation.

Because most Chinese patients cannot tolerate the high‐intensity statin treatment recommended by the ACC/AHA,^8^ atorvastatin 20 mg once daily (QD) was selected, based on Chinese dyslipidaemia guidelines and the expected LDL‐C response in Chinese patients.^28^, ^29^, ^30^ More recent guidelines in China are consistent with this approach.^11^ At screening and at each subsequent visit, patients were instructed to maintain their diet, as well as any allowed therapy and exercise regimen. The last dose of evolocumab or placebo was given at week 8 for QM patients and at week 10 for Q2W patients. The final study visit was at week 12 for QM patients and a phone visit at week 14 for Q2W patients. Baseline covariates for subgroup analysis of co‐primary efficacy endpoints included statin therapy at study entry (yes vs no), age, sex, race, baseline LDL‐C, family history of premature coronary heart disease, baseline PCSK9, body mass index, hypertension, status as current smoker, ≥2 baseline CHD risk factors and triglycerides. Treatment assignment was blinded to the sponsor's study team, investigators, site staff and patients throughout the study and remained blinded until unblinding of the clinical database.

### 2.2 Study assessments

As in the global population, blood assessments of patients enrolled at centres in China included fasting levels of lipids (total cholesterol, LDL‐C, triglycerides, VLDL‐C, non‐HDL‐C and HDL‐c) at screening, at the end of the lipid‐stabilisation period, at day1 (baseline) and at weeks 2, 8, 10 and 12. ApoB100 was assessed at day 1 and at weeks 10 and 12. Fasting serum glucose (FSG) (Roche GLUC3) was assessed at screening, at the end of the lipid‐stabilisation period, at day 1 and at weeks 8 and 12. HbA1c was assessed at screening and at week 12 (VARIANT II; Bio‐Rad, Hercules, CA, USA). Serum lipids and ApoB100 were measured by Medpace (Cincinnati, Ohio, USA, and Leuven, Belgium).^31^ FSG and HbA1c were measured as part of chemistry by Q2 Solutions (Valencia, CA, USA, and Livingston, Scotland). All blood analytes were measured by a central laboratory and results of the lipid panel, as well as apolipoproteins after treatment initiation, remained blinded until unblinding of the clinical database. Adverse events (AEs) were coded using the Medical Dictionary for Regulatory Activities, version 20.1. Blood samples for assessment of anti‐evolocumab antibodies were assayed on day 1 and at week 12.^32^

### 2.3 Statistics

As in the global population, co‐primary endpoints were the percentage change from baseline in LDL‐C at week 12 and the percentage change from baseline in LDL‐C at the mean of weeks 10 and 12. The average of weeks 10 and 12 better reflects average LDL‐C reduction during the dosing interval with monthly dosing. Co‐secondary endpoints included, but were not limited to, change from baseline in LDL‐C and percentage change from baseline in non‐HDL‐C, ApoB100, total cholesterol, triglycerides, HDL‐C, VLDL‐C and achievement of target LDL‐C 1.8 mmol/L (<70 mg/dL). Safety endpoints included incidence of treatment‐emergent AEs, laboratory values and incidence of binding and neutralizing anti‐evolocumab antibodies.

This pre‐specified analysis presents study results for all randomized patients enrolled at Chinese centres who received at least one one dose of study drug, that is, the China full analysis set (CFAS). The efficacy of evolocumab compared with placebo in the CFAS concerning the co‐primary endpoints (percentage change from baseline in LDL‐C at week 12 and the mean of weeks 10 and 12) and the continuous co‐secondary endpoints was compared using repeated‐measures linear mixed‐effects models with terms for treatment group, a stratification factor (statin therapy at study entry [yes vs no]), scheduled visit and interaction of treatment with the scheduled visit. In the CFAS, each independent dose frequency (Q2W and QM) was allocated a significance level of 0.025 for the co‐primary endpoints.^33^ LDL‐C target achievement was assessed using the Cochran‐Mantel‐Haenszel test, adjusted by existence of statin therapy at study entry (yes vs no). Safety assessments were summarized descriptively. As part of the China analyses, a subgroup analysis of co‐primary endpoints was also performed for Chinese vs non‐Chinese patients in FAS. Baseline characteristics for subgroup analysis of co‐primary efficacy endpoints included existence of statin therapy at study entry, age, sex, baseline LDL‐C, baseline PCSK9 level, body mass index, hypertension, current smoking status, baseline coronary heart disease risk factors and baseline triglycerides.

## 3 RESULTS

### 3.1 Patients

**Figure 1:**
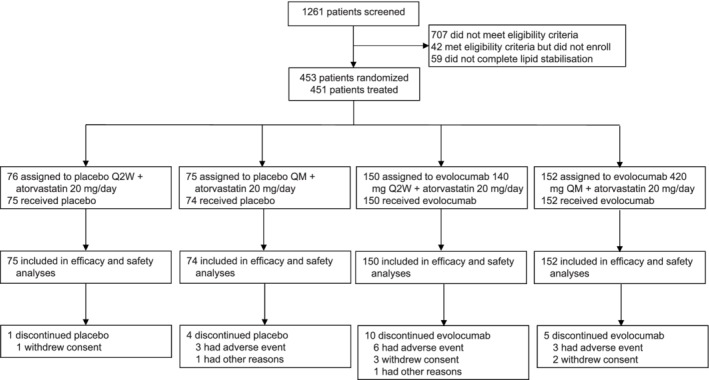
Patient enrollment and disposition

The study was conducted from 14 April 2016 through 6 December 2017. The global study population is described by Lorenzatti et al.^26^, ^27^ Overall, 1261 patients were screened at centres in China, of whom 453 were randomized. A total of 451 (99.6%) patients received at least one dose of study drug (the CFAS; evolocumab, n = 302; placebo, n = 149) and 431 (95.1%) patients completed the study (placebo, n = 144; evolocumab, n = 287) (Figure 1). Among the patients randomized, 451 (99.6%) patients (placebo, n = 150; evolocumab, n = 301) were using atorvastatin 20 mg/d at randomization, and 432 (95.4%) completed the study.

Demographics and baseline clinical characteristics of patients enrolled at centres in China were generally balanced between evolocumab and placebo groups, with 51.0% being female and with a mean age of 60.0 years (Table 1). As with the global population, baseline lipid values were similar in both treatment groups (Table 1). Consistent with the global population, there was an imbalance at baseline between the evolocumab and placebo groups in median HbA1c (median [Q1, Q3] 7.4% [6.7, 8.3] vs 7.0% [6.5, 7.9]), FSG (7.6 [6.5, 9.3] vs 7.1 [6.1, 8.8] mmol/L) and insulin use (45.7% vs 33.6%).

**Table 1:** Demographics and baseline characteristics and lipid values

Characteristics	Placebo (n = 149)	Evolocumab (n = 302)
Baseline demographics		
Median age (range), y	60 (35‐79)	61 (33‐79)
Sex, n (%)		
Female	81 (54.4)	149 (49.3)
Race, n (%)		
Asian	149 (100)	302 (100)
Baseline clinical characteristics		
Mean systolic blood pressure (SD), mm Hg	128.9 (14.8)	128.1 (14.2)
Mean body mass index (SD), kg/m^2^	25.3 (3.4)	25.7 (3.0)
Mean waist circumference (SD), cm	90.8 (10.7)	92.3 (8.5)
Statin therapy, n (%)	59 (39.6)	116 (38.4)
Intensive statin use^a^	3 (2.0)	8 (2.6)
Non‐intensive statin use^b^	56 (37.6)	108 (35.8)
Hypertension, n (%)	97 (65.1)	182 (60.3)
Cardiovascular disease^c^ n (%)	74 (49.7)	133 (44.0)
Cerebrovascular or peripheral arterial disease, n (%)	38 (25.5)	79 (26.2)
Coronary artery disease, n (%)	46 (30.9)	84 (27.8)
Baseline diabetes‐related medication use, n (%)	148 (99.3)	299 (99.0)
Insulin use, n (%)	50 (33.6)	138 (45.7)
Mean baseline lipid values (SD)		
LDL‐C, mmol/L	2.2 (0.8)	2.3 (0.9)
Non‐HDL‐C, mmol/L	2.9 (0.9)	3.0 (0.9)
ApoB100, g/L	0.81 (0.24)	0.83 (0.22)
Total cholesterol, mmol/L	4.2 (0.9)	4.2 (1.0)
Triglycerides, mmol/L	1.6 (1.8)	1.5 (0.7)
HDL‐C, mmol/L	1.2 (0.4)	1.2 (0.3)
VLDL‐C, mmol/L	0.70 (0.46)	0.68 (0.30)
Lp(a), nmol/L	64.0 (81.5)	65.6 (92.8)
Median baseline glucose metabolism measures (Q1, Q3)		
Haemoglobin A1c, %	7.00 (6.50, 7.90)	7.40 (6.70, 8.30)
Fasting plasma glucose, mmol/L	7.10 (6.10, 8.80)	7.60 (6.45, 9.25)

Abbreviations: ACC/AHA, American College of Cardiology/American Heart Association; ApoB100, apolipoprotein B100; HDL‐C, high‐density lipoprotein cholesterol; LDL‐C, low‐density lipoprotein cholesterol; Lp(a), lipoprotein(a); SD, standard deviation; VLDL‐C, very low‐density lipoprotein cholesterol.

a Patient had at least one of the following recorded for the last four weeks before screening: atorvastatin ≥40 mg QD; rosuvastatin ≥20 mg QD; simvastatin ≥80 mg QD (simvastatin 80 mg QD is not approved in some countries, eg, USA); and any statin (atorvastatin, fluvastatin, lovastatin, pitavastatin, pravastatin, rosuvastatin and simvastatin) QD plus ezetimibe.

b Patient has been taking any dose of a statin at least weekly for the last four weeks before screening and was not included in the intensive statin usage.

c Includes coronary artery disease, cerebrovascular disease and peripheral artery disease.

### 3.2 Efficacy

**Figure 2:**
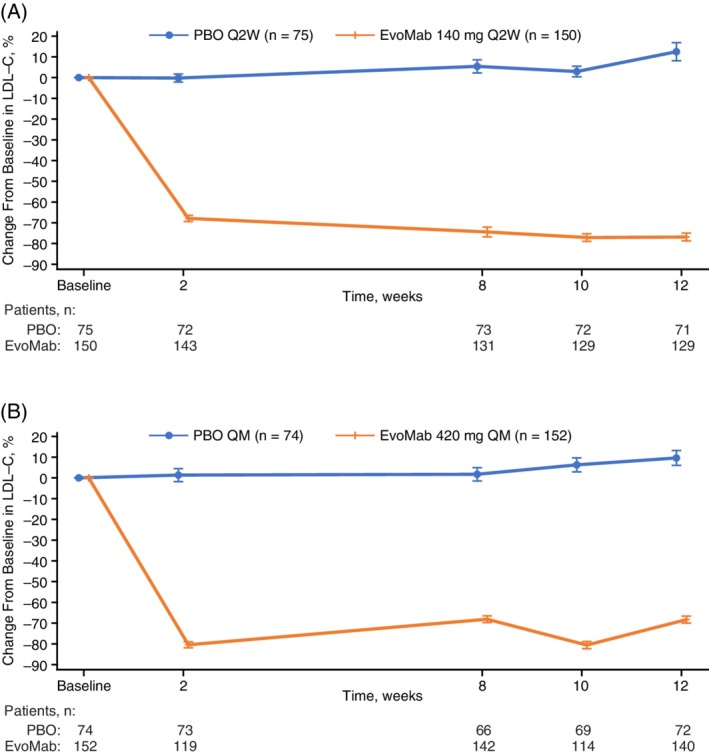
Mean percentage change in LDL‐C from baseline to scheduled visits in LDL‐C for Q2W dosing (A) and QM dosing (B). Vertical lines represent standard error around the mean. No imputation was used for missing values. When the calculated LDL‐C was <1.0 mmol/L or triglycerides were > 4.5 mmol/L, calculated LDL‐C was replaced with ultracentrifugation LDL‐C from the same blood sample, if available

Compared with placebo, evolocumab treatment reduced LDL‐C by a least‐squares mean of 85.0% (95% CI, −92.7 to −77.3; adjusted *P* < 0.0001) with Q2W dosing and 74.8% (−81.3 to −68.4; adjusted *P* < 0.0001) with QM dosing at week 12, and by 80.4% (−86.8 to −73.9; adjusted *P* < 0.0001) with Q2W dosing and 81.0% (−86.4 to −75.6; *P* < 0.0001) with QM dosing at the mean of weeks 10 and 12 (Table 2). At week 12, the least‐squares mean absolute change (95% CI) in LDL‐C was 0.23 mmol/L (0.06, 0.40) with placebo Q2W and −1.65 mmol/L (−1.77, −1.53) with evolocumab Q2W, and 0.14 mmol/L (0.001, 0.29) with placebo QM and −1.55 mmol/L (−1.65, −1.45) with evolocumab QM. At the mean of weeks 10 and 12, the least‐squares mean absolute change (95% CI) in LDL‐C was 0.15 mmol/L (−0.003, 0.31) with placebo Q2W and −1.63 mmol/L (−1.74, −1.52) evolocumab Q2W, and 0.12 mmol/L (−0.02, 0.25) with placebo QM and −1.72 mmol/L (−1.81, −1.62) with evolocumab QM. The treatment effect on LDL‐C, by scheduled visits and treatment groups for Q2W and QM regimens, is shown in Figure 2. LDL‐C concentrations were reduced to below 70 mg/dL (1.8 mmol/L) in 96.4% and 95.1% of patients in the evolocumab Q2W and QM groups, respectively, at week 12 and in 97.2% and 95.3% of patients in the evolocumab Q2W and QM groups, respectively, at the mean of weeks 10 and 12 (Table 2). Subgroup analyses of co‐primary endpoints demonstrated consistency of the effect of evolocumab treatment across all subgroups among Chinese patients (Figure S1). Trough evolocumab concentrations were comparable at weeks 8, 10 and 12 following administration of evolocumab 140 mg Q2W, and were comparable at weeks 8 and 12 in the evolocumab 420 mg QM treatment group (Table S1).

**Table 2:** Efficacy results at week 12 and at the mean of weeks 10 and 12

	Week 12				Mean of Weeks 10 and 12			
Parameter	PBO Q2W (n = 75)	EvoMab 140 mg Q2W (n = 150)	PBO QM (n = 74)	EvoMab 420 mg QM (n = 152)	PBO Q2W (n = 75)	EvoMab 140 mg Q2W (n = 150)	PBO QM (n = 74)	EvoMab 420 mg QM (n = 152)
LDL‐C								
n	72	137	72	144	74	143	72	148
Least squares mean change from baseline (SE), %	12.0 (3.2)	−73.0 (2.3)	9.5 (2.7)	−65.4 (1.9)	8.4 (2.7)	−72.0 (1.9)	7.8 (2.3)	−73.2 (1.6)
Mean treatment difference^a^ (SE), %	‐	−85.0 (3.9)	‐	−74.8 (3.3)	‐	−80.4 (3.3)	‐	−81.0 (2.7)
95% CI	‐	−92.7, −77.3	‐	−81.3, −68.4	‐	−86.8, −73.9	‐	−86.4, −75.6
Adjusted *P* value^b^	‐	< 0.0001	‐	< 0.0001	‐	< 0.0001	‐	< 0.0001
Achievement of 1.8 mmol/L, n (%)	17 (23.6)	132 (96.4)	18 (25.0)	137 (95.1)	18 (24.3)	139 (97.2)	17 (23.6)	141 (95.3)
Least squares mean change from baseline for secondary endpoints (SE), %								
Non‐HDL‐C	9.2 (2.9)	−61.1 (2.1)	6.7 (2.3)	−56.2 (1.6)	7.1 (2.5)	−61.0 (1.8)	5.5 (2.0)	−63.1 (1.4)
ApoB100	7.0 (2.3)	−58.0 (1.7)	3.8 (2.0)	−51.4 (1.4)	4.7 (2.0)	−58.1 (1.5)	2.0 (1.7)	−59.0 (1.2)
Total cholesterol	6.9 (2.1)	−40.4 (1.5)	5.0 (1.7)	−36.7 (1.2)	4.9 (1.9)	−40.8 (1.3)	3.8 (1.5)	−41.9 (1.0)
Lp(a)	2.2 (3.7)	−48.3 (2.7)	0.9 (3.6)	−42.1 (2.6)	−0.1 (3.1)	−49.0 (2.3)	0.4 (3.1)	−48.3 (2.2)
Triglycerides	3.6 (5.6)	−5.4 (4.1)	7.1 (3.7)	−11.1 (2.6)	6.9 (5.5)	−4.9 (4.0)	5.4 (3.7)	−13.1 (2.6)
HDL‐C	3.7 (1.9)	9.2 (1.4)	2.6 (1.9)	12.4 (1.3)	1.8 (1.6)	7.8 (1.2)	1.8 (1.8)	11.3 (1.2)
VLDL‐C	2.5 (4.2)	−12.1 (3.1)	7.1 (3.9)	−13.6 (2.8)	5.4 (4.3)	−17.4 (3.1)	6.5 (3.6)	−21.4 (2.5)

Abbreviations: ApoB100, apolipoprotein B100; CI, confidence interval; HDL‐C, high‐density lipoprotein cholesterol; LDL‐C, low‐density lipoprotein cholesterol; Lp(a), lipoprotein(a); SE, standard error; VLDL‐C, very low‐density lipoprotein cholesterol.

a Treatment difference is from the repeated measures linear effects model, which included treatment group, stratification factors, scheduled visit and interaction of treatment with scheduled visit as covariates for all endpoints except LDL‐C achievement.

b Adjusted *P* value is based on a combination of sequential testing, the Hochberg procedure, which is a fallback procedure to control the overall significance level for all primary and secondary endpoints. Each individual adjusted *P* value is compared to 0.05 to determine statistical significance.

**Figure 3:**
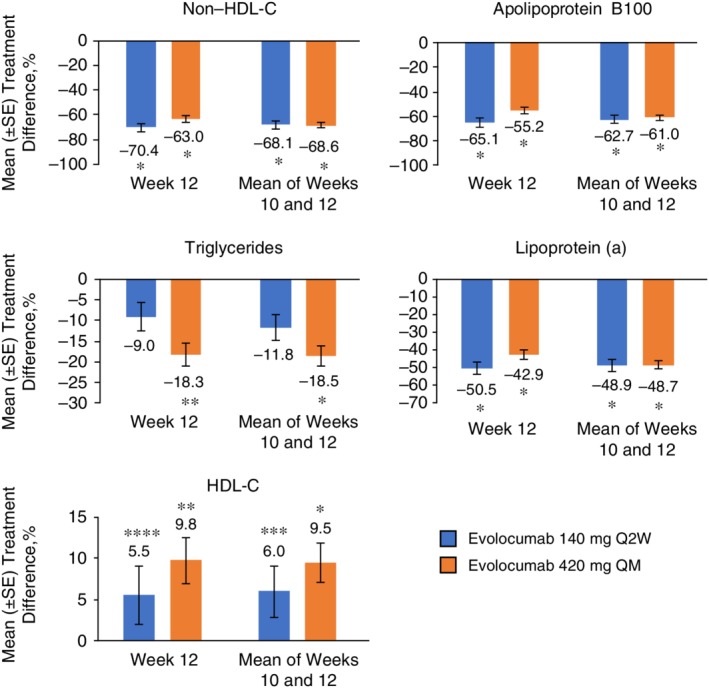
Mean percentage treatment difference for evolocumab vs placebo in non‐HDL‐C, apolipoprotein B100, triglycerides, lipoprotein (a) and HDL‐C. Treatment difference is from the repeated measures linear effects model, which included treatment group, stratification factors, scheduled visit and interaction of treatment with scheduled visit as covariates for all endpoints except LDL‐C achievement. Error bars show standard error Abbreviations: HDL‐C, high‐density lipoprotein cholesterol. ^*^ *P* < 0.0001 vs placebo; ^**^ *P* = 0.0002 vs placebo; ^***^ *P* = 0.003 vs placebo; ^****^ *P* = 0.033 vs placebo

Changes from baseline in secondary lipid parameters are summarized in Table 2. Compared with placebo, treatment with evolocumab resulted in statistically significant improvements in non‐HDL‐C from baseline to week 12 (Q2W, −70.4%; adjusted *P* < 0.0001; QM, −63.0%; adjusted *P* < 0.0001) and to the mean of weeks 10 and 12 (Q2W, −80.4%; adjusted *P* < 0.0001; QM, −81.0%; adjusted *P* < 0.0001) (Figure 3). Treatment with evolocumab resulted in statistically significant improvements in the least squares mean (SD) percent change from baseline in Lp(a) (from baseline to week 12, Q2W: −48.3% [2.7%]; QM: −42.1% [2.6%]; to the mean of weeks 10 and 12, Q2W: −49.0% [2.3%]; QM: −48.3 [2.2%]; *P* < 0.0001). Treatment with evolocumab also resulted in significant improvements in ApoB100, triglycerides and HDL‐C.

Both co‐primary endpoints demonstrated statistically significant reductions in LDL‐C with evolocumab Q2W and QM compared with placebo, for both non‐Chinese and Chinese subgroups. At the mean of weeks 10 and 12, compared with placebo, the estimated treatment difference in reduction in LDL‐C among Chinese vs non‐Chinese participants was 19.0% (95% CI, 8.9%‐29.0%; adjusted *P* < 0.0002) for evolocumab Q2W and was 20.3% (11.4%‐29.3%; adjusted *P* < 0.0001) for evolocumab QM. At week 12, the corresponding difference in LDL‐C reduction was 25.1% (95% CI, 13.7%‐36.5%; adjusted *P* < 0.0001) for evolocumab Q2W and was 18.2% (8.4%‐28.1%; adjusted *P* < 0.0003) for evolocumab QM. For this subgroup analysis, qualitative interaction testing demonstrated common directionality of the LDL‐C lowering effect and non‐significant *P*‐values.

### 3.3 Safety

Among patients enrolled at Chinese centres, the incidence of AEs was greater in the overall evolocumab group than in the overall placebo group (57.6% vs 53.0%) (Table 3). Most were mild, grades 1 (placebo, 16.1%; evolocumab, 18.2%) or 2 (placebo, 30.2%; evolocumab, 28.5%). The most common AEs in the overall evolocumab group were upper respiratory tract infection, diabetes mellitus (described as worsening or exacerbation of pre‐existing diabetes), and nasopharyngitis.

**Table 3:** Incidence of treatment‐emergent adverse events

Characteristics	Placebo (n = 149)	Evolocumab (n = 302)
Patients with AEs, n (%)	79 (53.0)	174 (57.6)
Patients with serious AEs, n (%)	6 (4.0)	24 (7.9)
Patients with AEs leading to treatment discontinuation, n (%)	2 (1.3)	9 (3.0)
Fatal AEs, n (%)	0	0
Common AEs^a^, n (%)		
Diabetes mellitus^b^	3 (2.0)	22 (7.3)
Upper respiratory tract infection	11 (7.4)	20 (6.6)
Nasopharyngitis	9 (6.0)	15 (5.0)
Dizziness	2 (1.3)	8 (2.6)
Urinary tract infection	3 (2.0)	7 (2.3)
Blood uric acid increased	3 (2.0)	7 (2.3)
Hypertension	4 (2.7)	7 (2.3)
Headache	0	6 (2.0)
Blood glucose increased	0	5 (1.7)
Palpitations	0	5 (1.7)
Cough	4 (2.7)	4 (1.3)
Toothache	0	4 (1.3)
Back pain	3 (2.0)	4 (1.3)
Hyperuricaaemia	1 (0.7)	4 (1.3)
Atrial fibrillation	1 (0.7)	4 (1.3)
Peripheral oedema	0	4 (1.3)
Median change from baseline in glycaemic measures (Q1, Q3)		
Haemoglobin A1c, %	0.2 (−0.1, 0.6)	0.2 (−0.3, 0.8)
Fasting serum glucose, mmol/L	0.1 (−0.4, 0.9)	0.1 (−0.7, 1.2)
Abnormal laboratory tests, n (%)		
Creatine kinase >5× ULN	1 (0.7)^c^	0
Alanine aminotransferase >3 × ULN	1 (0.7)	0
Aspartate aminotransferase >3 × ULN	1 (0.7)	0

Abbreviations: AE, adverse event; ULN, upper limit of normal.

a Occurring in at least 1% of patients in the evolocumab treatment group.

b Includes preferred terms of diabetes mellitus (placebo, 2.0%; evolocumab, 5.6%) and type 2 diabetes mellitus (placebo, 0%; evolocumab, 1.7%).

c Patient was assigned to QM dosing, reported at week 8 visit, remained asymptomatic, and returned to baseline at week 12.

Most diabetes mellitus AEs were mild in severity (grade 1 or 2) and were managed with medication and/or diet and exercise counselling. As in the global population, no notable changes from baseline in glycaemic parameters were observed during the study, as median (Q1, Q3) changes from baseline to week 12 in HbA1c were 0.2% (−0.3, 0.8) in the evolocumab group and 0.2% (−0.1, 0.6) in the placebo group, and median changes from baseline to week 12 in FSG were 0.1 mmol/L (−0.7, 1.15) in the evolocumab group and 0.1 mmol/L (−0.4, 0.9) in the placebo group (Table 3).

Serious AEs occurred in 24 (7.9%) patients in the overall evolocumab group and in six (4.0%) patients in the overall placebo group. No single serious AEs were reported in more than 1% of patients who received evolocumab or placebo. Serious AEs were not considered by the investigators to be related to the study drug, with exception of gastric ulcer (n = 1, evolocumab) and lacunar stroke (n = 1, evolocumab). No fatal AEs occurred during the study. No patients tested positive for anti‐evolocumab binding or neutralizing antibodies.

## 4 DISCUSSION

To our knowledge, the BERSON study is the first dedicated T2DM study involving a PCSK9 inhibitor that enrolled patients in China. In this pre‐specified analysis of the BERSON study, compared with placebo, patients with T2DM and hyperlipidaemia or mixed dyslipidaemia who received evolocumab Q2W or QM with background atorvastatin at Chinese centres had significant reductions in LDL‐C, by 75% to 85%, and in non‐HDL‐C, by 63% to 81%. Significant improvements in other atherogenic lipid parameters were also observed after 12 weeks of treatment with evolocumab. Furthermore, more than 90% of the Chinese patients who received evolocumab with background atorvastatin of moderate intensity were able to achieve LDL‐C values below 1.8 mmol/L (95%‐97% with evolocumab vs 24%‐25% with placebo). These results are noteworthy, considering that, in previous studies in Chinese and Southeast Asian community hospitals or clinics, the majority of patients with diabetes and CVD risk factors who received statins or other lipid‐lowering therapies did not attain recommended levels of LDL‐C.^15^, ^16^, ^17^, ^18^ While subgroup analysis of co‐primary endpoints showed statistically significant differences in the magnitude of LDL‐C reduction between Chinese and non‐Chinese populations, treatment effects are directionally consistent, favouring evolocumab, in the two subgroups. Least‐squares mean Lp(a) reduction with evolocumab, a co‐secondary efficacy endpoint, was also greater in Chinese patients compared with the global population. These findings could not be explained by differences in adherence to the investigational product or to other lipid‐lowering therapies, by LDL levels in the placebo groups or by differences in evolocumab pharmacokinetic exposure or serum PCSK9 levels; thus, further investigation may be warranted. Observations in the Chinese population are consistent with results reported in Japanese patients treated with evolocumab over 12 weeks, with least‐squares mean LDL‐C reductions ranging from 67% to 76%, compared with placebo, when combined with atorvastatin 20 mg daily, and LDL‐C less than 1.8 mmol/L was achieved by 96% to 98% of patients.^34^

The substantial reductions in LDL‐C with evolocumab vs placebo among Chinese patients in the BERSON study are consistent with those observed in prior evolocumab global studies in patients with hypercholesterolaemia or mixed dyslipidaemia,^35^ in patients with T2DM,^36^ and in a meta‐analysis and pooled analysis of data from 12‐week phase 3 studies.^24^, ^37^ Prior analyses showed that the LDL‐C treatment effect with evolocumab is maintained over time in patients with and without diabetes.^21^, ^23^ Our results are also consistent with those from a systematic review including 20 randomized controlled studies with PCSK9 inhibitors (alirocumab, bococizumab and evolocumab).^38^ The ODYSSEY KT study, which assessed the efficacy and safety of the PCSK9 inhibitor alirocumab in 199 patients at high cardiovascular risk enrolled in South Korea and Taiwan, also showed statistically significant reductions in LDL‐C.^39^ At week 12, alirocumab 75 mg Q2W reduced LDL‐C by a least‐squares mean of 57.9% (standard error [SE], 2.2%) vs placebo, which increased by 4.7% (SE, 2.2%). At week 24, alirocumab reduced LDL‐C by a least‐squares mean of 57.1% (SE, 3.05) vs placebo, which increased by 6.3% (2.9%). The phase 3 ODYSSEY Japan study in high‐risk patients udergoing stable statin therapy showed consistent LDL‐C reduction sustained to week 52.^40^

Evolocumab, combined with background moderate intensity atorvastatin, was well tolerated among patients in the BERSON study who enrolled at centres in China. Most serious AEs were not considered to be related to treatment with evolocumab. In both the overall evolocumab group and the placebo group, patients in China had a greater incidence of AEs overall (evolocumab, 57.6% vs 43.8%; placebo, 53.0% vs 42.6%), of serious AEs (evolocumab, 7.9% vs 4.9%; placebo, 4.0% vs 3.0%) and frequently occurring AEs, including upper respiratory tract infection (evolocumab, 6.6% vs 4.6%; placebo, 7.4% vs 1.9%), of diabetes (evolocumab, 5.6% vs 4.6%; placebo, 2.0% vs 1.9%) and of nasopharyngitis (evolocumab, 5.0% vs 3.0%; placebo, 6.0% vs 3.7%) compared with the global BERSON study population.^24^ The reason for this difference between patients enrolled in China and in other countries is not clear. However, there were no noteworthy differences between the global population^41^ and the Chinese population in terms of type or severity of the most frequently reported AEs and no noteworthy differences between the evolocumab and placebo treatment groups in either population, and the overall safety profile of evolocumab in Chinese patients was similar to that reported in previous global studies of evolocumab.^23^, ^25^, ^37^, ^42^

As in the global population, although AEs of diabetes occurred more often in the evolocumab treatment group than in the placebo group (5.6% vs 2.0%) no notable changes in glycaemic control (HbA1c and FPG) in patients at Chinese centres were noted between treatment groups, and most events were mild (grades 1 or 2) in severity. The magnitude of difference in the AEs of diabetes among patients who received evolocumab was not observed in prior evolocumab studies and could be attributed, at least in part, to inferior diabetes control at baseline in the evolocumab group as compared to the placebo group, as reflected in the imbalance observed in HbA1c (7.4% vs 7.0%), in FPG (7.6 vs 7.1 mmol/L) and in insulin use (45.7% vs 33.6%), respectively.^21^, ^23^, ^25^, ^43^ Results from prior studies with alirocumab showed no impact on glycaemic parameters or on the risk of new‐onset diabetes.^39^, ^44^, ^45^, ^46^ A meta‐analysis of 18 studies in patients without diabetes (N = 26 123) showed no difference in new‐onset diabetes, fasting glucose or HbA1c between patients who received a PCSK9 inhibitor (alirocumab or evolocumab) or control patients.^47^

Although this study was limited to a 12‐week follow‐up period, the efficacy and safety results are consistent with those of prior prespecified and *post hoc* analyses of evolocumab in patients with T2DM that have a larger sample size and/or a longer duration.^23^, ^24^ An additional limitation is the use of a non‐intensive statin (atorvastatin 20 mg/d) that is more conservative than that in other regions and limits comparison of these results with other global studies.

In conclusion, in this population of patients from the BERSON study with T2DM and hyperlipidaemia or mixed dyslipidaemia who were enrolled in China, treatment with evolocumab, combined with background atorvastatin, significantly reduced LDL‐C and other atherogenic lipid parameters. Evolocumab was safe and well tolerated, without affecting measures of glycaemic control.

## CONFLICTS OF INTEREST

F. G. E. has served as a speaker for and has received grants for research from Amgen Inc., Sanofi, Boehringer, Eli Lilly, Novo Nordisk and AstraZeneca. A. J. L. has served as an advisory board and steering committee member for and has received research grants and speaker fees from Amgen Inc. M. L. M., N. W. and A. W. H. are employed by and own stock in Amgen Inc. Y. C., Z. Y., J. L. and J. G. have no conflicts of interest to disclose.

## AUTHOR CONTRIBUTIONS

Y. C., Z. Y., J. L. and J. G**.** acquired data for the study. F. G. E., A. J. L, M. L. M, N. W. and A. W. H acquired and interpreted the data. All authors are responsible for the work described in this paper. All authors either drafted the manuscript or critically reviewed the manuscript for important intellectual content. All authors gave final approval of the version to be published and agree to be accountable for all aspects of the work, ensuring that questions related to the accuracy or integrity of any part of the work are appropriately investigated and resolved. Qualified researchers may request data from Amgen clinical studies. Complete details are available at: http://www.amgen.com/datasharing.

## Supporting information


**Table S1** Serum evolocumab concentrations in the global and China analyses of the BERSON study.
**Figure S1**. Treatment differences in percentage change from baseline in LDL‐C at week 12 in Chinese patient subgroups. BMI, body mass index; CHD, coronary heart disease; CI, confidence interval; EvoMab, evolocumab; LDL‐C, low‐density lipoprotein cholesterol; n1, number of patients in the subgroup of interest with an observed value at each dose frequency receiving EvoMab; n2, number of patients in the subgroup of interest with an observed value at each dose frequency receiving placebo; PCSK9, proprotein convertase subtilisin/kexin type 9; Q2W, every 2 weeks; QM, monthly. When the calculated LDL‐C was <40 mg/dL or triglycerides were > 400 mg/dL, calculated LDL‐C was replaced with ultracentrifugation LDL‐C from the same blood sample, if available. Least squares mean differences and 95% CIs are from the repeated measures model. No imputation was used for missing values. In these subgroup analyses, CHD risk factors are the same as the cardiovascular risk factors described in the baseline disease characteristics.
