# Supplementary material for: Randomized study of evolocumab in patients with type 2 diabetes and dyslipidaemia on background statin: Pre‐specified analysis of the Chinese population from the BERSON clinical trial
Source: Diabetes Obes Metab. 2019 Apr 14;21(6):1464–73. doi: 10.1111/dom.13700 (PMC6594089; doi:10.1111/dom.13700)

**Supplemental Table 1.** **Serum evolocumab concentrations in the global and China analyses of the BERSON study.**

| Population | Day 1 | Week 2 | Week 8 | Week 10 | Week 12 |
| --- | --- | --- | --- | --- | --- |
| Global Q2W | 0.0126 (0.226) | 1.44 (2.43) | 4.33 (5.11) | 4.09 (5.30) | 4.22 (4.57) |
| China Q2W | 0 | 1.22 (2.59) | 3.46 (3.41) | 3.34 (2.95) | 3.78 (3.44) |
| Global QM | 0.0475 (0.446) | 21.4 (12.8) | 6.14 (7.24) | 27.5 (17.8) | 7.11 (8.29) |
| China QM | 0 | 21.4 (11.6) | 6.04 (5.64) | 27.8 (14.5) | 6.65 (6.15) |

Values are mean [SD] concentration in µg/mL

**Supplemental Figure 1**. Treatment differences in percentage change from baseline in LDL-C at week 12 in Chinese patient subgroups. BMI, body mass index; CHD, coronary heart disease; CI, confidence interval; EvoMab, evolocumab; LDL-C, low-density lipoprotein cholesterol; n1, number of patients in the subgroup of interest with an observed value at each dose frequency receiving EvoMab; n2, number of patients in the subgroup of interest with an observed value at each dose frequency receiving placebo; PCSK9, proprotein convertase subtilisin/kexin type 9; Q2W, every 2 weeks; QM, monthly. When the calculated LDL-C was <40 mg/dL or triglycerides were >400 mg/dL, calculated LDL-C was replaced with ultracentrifugation LDL-C from the same blood sample, if available. Least squares mean differences and 95% CIs are from the repeated measures model. No imputation was used for missing values. In these subgroup analyses, CHD risk factors are the same as the cardiovascular risk factors described in the baseline disease characteristics.


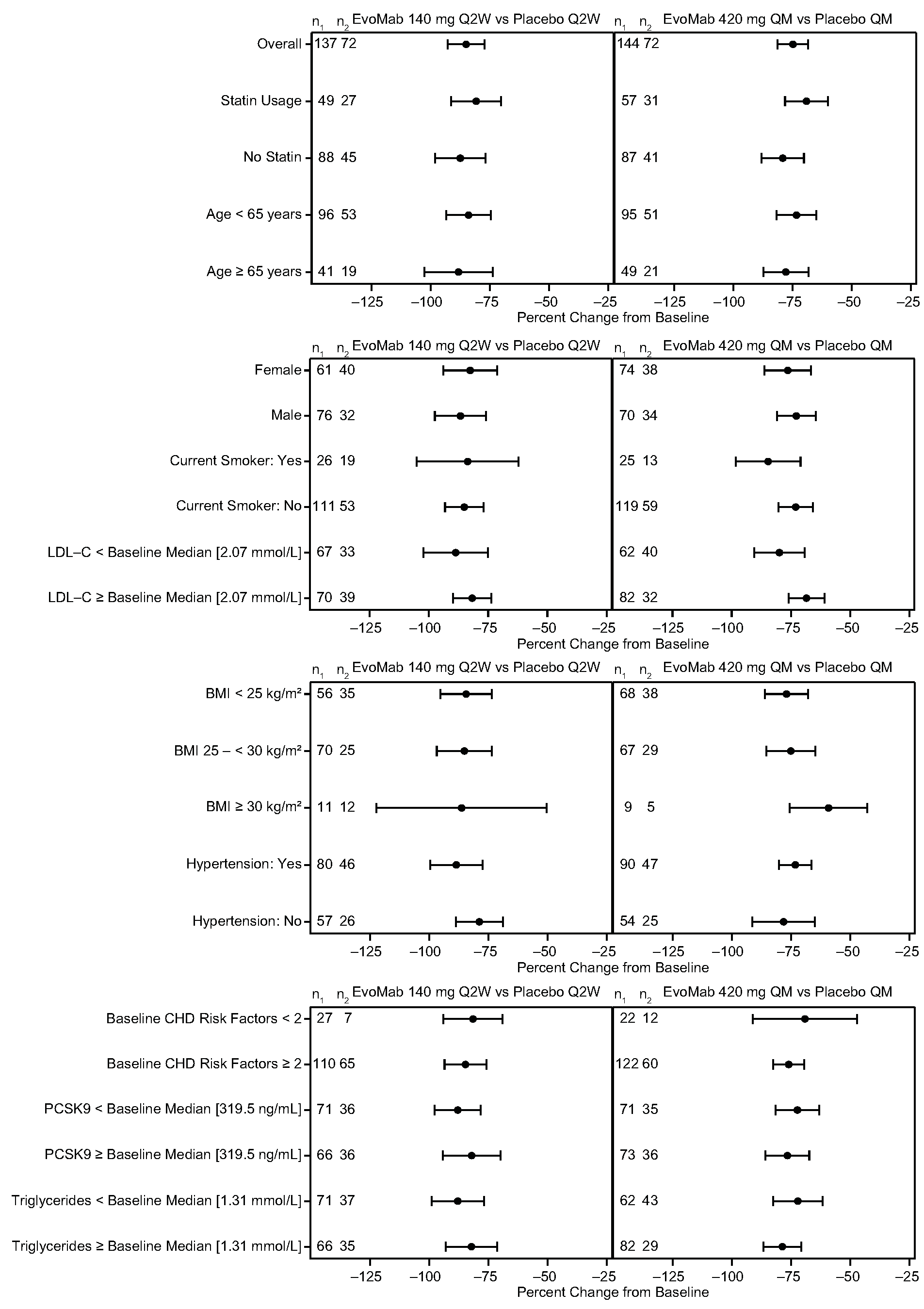

Supplement: Supplementary file 1 — Table S1 Serum evolocumab concentrations in the global and China analyses of the BERSON study. Figure S1. Treatment differences in percentage change from baseline in LDL‐C at week 12 in Chinese patient subgroups. BMI, body mass index; CHD, coronary heart disease; CI, confidence interval; EvoMab, evolocumab; LDL‐C, low‐density lipoprotein cholesterol; n1, number of patients in the subgroup of interest with an observed value at each dose frequency receiving EvoMab; n2, number of patients in the subgroup of interest with an observed value at each dose frequency receiving placebo; PCSK9, proprotein convertase subtilisin/kexin type 9; Q2W, every 2 weeks; QM, monthly. When the calculated LDL‐C was <40 mg/dL or triglycerides were > 400 mg/dL, calculated LDL‐C was replaced with ultracentrifugation LDL‐C from the same blood sample, if available. Least squares mean differences and 95% CIs are from the repeated measures model. No imputation was used for missing values. In these subgroup analyses, CHD risk factors are the same as the cardiovascular risk factors described in the baseline disease characteristics. [file DOM-21-1464-s001.docx]
